# Single-cell multi-omics analysis reveals dysfunctional Wnt signaling of spermatogonia in non-obstructive azoospermia

**DOI:** 10.3389/fendo.2023.1138386

**Published:** 2023-06-06

**Authors:** Shengjie Zeng, Liuxun Chen, Xvdong Liu, Haibin Tang, Hao Wu, Chuan Liu

**Affiliations:** ^1^ Department of Urology, The Second Affiliated Hospital of Chongqing Medical University, Chongqing, China; ^2^ Department of Cardiothoracic Surgery, The First Affiliated Hospital of Chongqing Medical University, Chongqing, China; ^3^ Department of Urology, The First Affiliated Hospital of Chongqing Medical University, Chongqing, China

**Keywords:** non-obstructive azoospermia, ScRNA-seq, scATAC-seq, spatial transcriptomic data, Wnt signaling pathway, CCCTC-binding factor (CTCF), androgen receptor (AR), ARNTL

## Abstract

**Background:**

Non-obstructive azoospermia (NOA) is the most severe type that leads to 1% of male infertility. Wnt signaling governs normal sperm maturation. However, the role of Wnt signaling in spermatogonia in NOA has incompletely been uncovered, and upstream molecules regulating Wnt signaling remain unclear.

**Methods:**

Bulk RNA sequencing (RNA-seq) of NOA was used to identify the hub gene module in NOA utilizing weighted gene co-expression network analyses (WGCNAs). Single-cell RNA sequencing (scRNA-seq) of NOA was employed to explore dysfunctional signaling pathways in the specific cell type with gene sets of signaling pathways. Single-cell regulatory network inference and clustering (pySCENIC) for Python analysis was applied to speculate putative transcription factors in spermatogonia. Moreover, single-cell assay for transposase-accessible chromatin sequencing (scATAC-seq) determined the regulated genes of these transcription factors. Finally, spatial transcriptomic data were used to analyze cell type and Wnt signaling spatial distribution.

**Results:**

The Wnt signaling pathway was demonstrated to be enriched in the hub gene module of NOA by bulk RNA-seq. Then, scRNA-seq data revealed the downregulated activity and dysfunction of Wnt signaling of spermatogonia in NOA samples. Conjoint analyses of the pySCENIC algorithm and scATAC-seq data indicated that three transcription factors (*CTCF*, *AR*, and *ARNTL*) were related to the activities of Wnt signaling in NOA. Eventually, spatial expression localization of Wnt signaling was identified to be in accordance with the distribution patterns of spermatogonia, Sertoli cells, and Leydig cells.

**Conclusion:**

In conclusion, we identified that downregulated Wnt signaling of spermatogonia in NOA and three transcription factors (*CTCF*, *AR*, and *ARNTL*) may be involved in this dysfunctional Wnt signaling. These findings provide new mechanisms for NOA and new therapeutic targets for NOA patients.

## 1 Introduction

One in seven couples suffer from infertility globally, and almost half of them are ascribed to male factors. Up to 15% of infertile men are caused by azoospermia, which is characterized by the non-existence of sperm in semen (1). The most severe type of azoospermia is non-obstructive azoospermia (NOA), which is defined as the absence of sperm in the ejaculate due to impaired spermatogenesis (2). Although the etiologies of NOA are largely unknown, impairment in any steps of spermatogenesis can lead to NOA (3). Spermatogonia serve as the fuel of spermatogenesis and the precursors of all sperm; therefore, abnormality in the proliferation and differentiation of spermatogonia can cause NOA (4). In addition, therapies targeted toward spermatogonia are becoming an increasingly used treatment for NOA (5). Takehiko et al. successfully used spermatogonial transplantation to restore the fertility of infertile mouse strains (6). Spermatogonial transplantation is a promising way to treat NOA. Thus, investigations into the aberrant genetic and epigenetic events in spermatogonia are essential to improve the safety of spermatogonial transplantation and find new therapeutic targets for NOA.

There are substantial signal pathways involved in the onset of NOA. It has been shown that the Wnt signaling pathway, as an important pathway for cell growth and development, plays an instrumental role in mammalian spermatogenesis (7). It is well documented that Wnt signaling governs sperm maturation (8). However, the role of Wnt signaling in spermatogonia, as the fuel of sperm, is not thoroughly explored. More importantly, no multi-omics analyses were documented in the studies that aimed to investigate the role of Wnt signaling in spermatogonia (9). Thus, how the Wnt signaling pathway influences spermatogonia of NOA patients remains unknown.

Single-cell analysis sheds light on molecular data of a wide variety of diseases at an unprecedented resolution (10). Single-cell multi-omics analysis includes, but is not limited to, single-cell RNA sequencing (scRNA-seq), single-cell assay for transposase-accessible chromatin sequencing (scATAC-seq), and spatial transcriptomics analysis. ScRNA-seq revealed new cell types in the testis that promote spermatogenesis (11). ScATAC-seq is designed to study open chromatin in single cells, which identified key transcription factors during meiosis (12). Spatial transcriptomics analysis comprises spatial location information to compensate for the lost original tissue architecture information in other analyses.

Therefore, the aim of this study was to capture a comprehensive picture of the Wnt signaling in spermatogonia of NOA patients using multi-omics analysis. We obtained RNA-seq and scRNA-seq data of normal and NOA samples from a public database. Downregulated activity and dysfunction of the Wnt signaling pathway of spermatogonia in NOA samples were subsequently explored. In addition, a new gene co-expression network associated with the Wnt pathway was established. Single-cell regulatory network inference and clustering (pySCENIC) was used to infer the transcription factor (TF) of spermatogonia in normal and NOA samples. Subsequently, scATAC-seq demonstrated that three TFs were actively involved in the Wnt signaling pathway. Finally, spatial transcriptomic data validated cell-type specific Wnt signaling. Thus, these results indicate that the dysfunctional Wnt signaling pathway performs an important role in NOA and three TFs (CTCF, AR, and ARNTL), which may be involved in this process. These findings provide new mechanisms for NOA and new therapeutic targets for NOA patients.

## 2 Materials and methods

### 2.1 Data source

Bulk RNA-seq data of a total of 100 NOA samples and 33 normal samples (free from any major disease) were obtained from the GEO database (https://www.ncbi.nlm.nih.gov/geo/) (accession number: GSE45885, GSE9210, GSE108886, GSE145467, and GSE216907). The clinical features are listed in **Supplementary Table S4**. ScRNA-seq data of 7 NOA samples and 22 normal samples were accessed from the open-access data of Nie et al. (13) and Zhao et al. (14). The clinical information of used samples in scRNA-seq is presented in **Supplementary Tables S1**, **S2**. ScATAC-seq and spatial transcriptome dataset were acquired from publicly available data of Garcia-Alonso et al. (14). Gene sets for hallmarks of spermatogenesis and hallmarks of Wnt signaling were obtained from MsigDB hallmark gene sets (http://www.gsea-msigdb.org/gsea/msigdb).

### 2.2 Data procession

For bulk RNA-seq, the raw data were processed using the limma R package with the log-normalization method, based on |log2(fold change(FC)) > 1 and p < 0.01. As for scRNA-seq data, the Seurat R package workflow was used for cell filtration, normalization, dimensionality reduction, and clustering. IntegrateData function based on the canonical-correlation analysis (CCA) algorithm was performed to eliminate batch effects. Perhaps because the downloaded data underwent quality control, 61,622 cells out of 68,066 cells remained according to the standard: minGene = 300, maxGene = 7,000, mitochondrial content <15%. Uniform manifold approximation and projection (UMAP) and resolution = 0.8 was selected to perform dimensionality reduction and clustering. Finally, cell type annotation was achieved by marker genes from previous research (15). For scATAC-seq data, the 10X Genomics Cell Ranger ATAC pipeline (v.1.2.0) with the GRCh38 genome was utilized as a reference. Then, the Signac package was used for downstream analysis. According to the standard of quality control, 750 cells in total were obtained: peak_region_fragments >1,000 reads and <20,000, pct reads in peaks >15, blacklist ratio <0.05, nucleosome_signal <4, and TSS.enrichment >1.2. Thereafter, the FindTransferAnchors function was conducted to annotate cell types of scATAC-seq according to the cell types of scRNA-seq based on the anchor points. For the spatial transcriptome dataset, we downloaded processed data treated with the Seurat package. Spots with less than 200 measured genes and less than 500 unique molecular identifiers (UMIs) were filtered out. Visualization of spatial transcriptome data was carried out using the Seurat package.

### 2.3 Unsupervised clustering and GO enrichment analysis

Unsupervised clustering was conducted with the ConsensusClusterPlus package. The Kuhn–Munkres (KM) algorithm was performed, and “euclidean” was used as measurement distance. Sampling using 1,000 bootstraps was conducted, with each bootstrap containing 80% samples, which yielded four predominant clusters. Differential genes of these four clusters were determined by comparing each cluster to the rest of the clusters based on the following criteria: |log2(FC)| > 1 and p < 0.01. Then, these differential genes were subjected to Gene Ontology (GO) enrichment analysis with the clusterProfiler package. The Benjamini–Hochberg method was employed to adjust the p-values (adjust-p <0.05 was considered significant).

### 2.4 Weighted gene co-expression network analysis

Weighted gene co-expression network analysis (WGCNA) is a powerful bioinformatics tool that builds gene network modules related to diseases by applying the R package WGCNA (16). A weighted adjacency matrix was generated by calculating Pearson’s correlation between each gene pair. The power of 8 was selected as a soft threshold parameter to classify genes into different gene modules with a minimum size of 30 genes. The top 200 genes with the highest node centrality, which was calculated by principal component analysis within each module, were extracted for subsequent analysis. An important objective of WGCNA is to identify the gene modules that are closely correlated with clinical phenotypes. To this end, the Bicor function of the WGCNA package was employed to analyze the correlation between phenotype and each gene module using the biweight midcorrelation (bicor) method.

### 2.5 Multiscale embedded gene co-expression network analysis

Multiscale Embedded Gene Co-Expression Network Analysis (MEGENA) is another method used in establishing gene hierarchy clustering, which shows improved performance over WGCNA (17). WGCNA and MEGENA are powerful methods for identifying significant genes. WGCNA has the limitation of not being able to coexist at different levels of clustering within a single network, thus not reflecting the multiscale hierarchical nature of complex networks. MEGENA, in contrast, allows the construction and analysis of large-scale planar filtered co-expression networks to the greatest extent possible. As performed in the study of Lei et al., we sought to combine both methods (18). Default parameters in the MEGENA R package were employed to perform MEGENA. A minimum module size of 100 was used to calculate a planar filtered network (PFN) from gene expression correlations. The multiscale clustering method was applied to build a glioma gene network consisting of interconnected subnetworks or modules (19). The genes in the MEGENA-C1_3 cluster were visualized using Cytoscape 3.7.2.

### 2.6 Gene set variation analysis and AUCell

Gene set variation analysis (GSVA) is a sensitive technique that identifies the enrichment of gene sets in samples using gene sets from MsigDB, GO database, and Kyoto Encyclopedia of Genes and Genomes (KEGG) database. GSVA has been used in scRNA-seq data in recent years (20). AUCell is another method to determine whether a given gene set is enriched in single cells (21). Since the computation of GSVA is less time-consuming, we applied GSVA to huge GO datasets with the GSVA package, while we employed AUCell analysis in HALLMARK_SPERMATOGENESIS and HALLMARK_WNT_BETA_CATENIN_SIGNALING gene sets from MsigDB with AUCell package, which represented sperm development in male fertility and canonical beta-catenin pathway separately (22).

### 2.7 Cell communication analysis

CellChat R package was run using its default settings to conduct cell communication analysis. The principle of CellChat is to evaluate the expression of receptor–ligand pairs from the CellChatDB database in different cell types to prove the existence of exact signaling pathways (23). The first step was downloading the receptor–ligand interactions from the CellChatDB database. Then, to evaluate whether different cell types had remarkable correlations, the marker genes from the source cell type were searched against the marker genes from the target cell type based on the receptor–ligand pairs in the CellChatDB. Subsequently, 10,000 permutations were implemented for the expressed genes to assess the background frequencies of receptor–ligand pairs.

### 2.8 High-dimensional weighted gene co-expression network analysis

High-dimensional weighted gene co-expression network analysis (hdWGCNA) is an R package that was especially developed for scRNA-seq identifying disease-relevant co-expression network modules. We followed typical hdWGCNA workflow. First, we removed batch effects between samples with a harmony algorithm. Next, we generated metacells with k = 200, and we screened the soft power threshold (threshold = 0.85, power = 12). Then, we constructed a topological matrix via the topological overlap matrix (TOM). We identified gene modules using module-cutting parameters, including minModuleSize = 50 and mergeCutHeight = 0.2.

### 2.9 Single-cell regulatory network inference and clustering for Python

To speculate putative dominant TFs in spermatogonia from scRNA-seq, we conducted pySCENIC with pySCENIC R package (24). In brief, we constructed a gene regulation network (TFs and known target genes) from scRNA-seq. Then, we analyzed the activity of each TF in single cells via the AUCell function of pySCENIC. The results of pySCENIC are presented in **Supplementary Table S3**. To identify main TFs in NOA or normal spermatogonia, we used the RSS method implemented in the calcRSS function in the R package SCENIC, which has been proven to be an effective method to identify cell type-specific TFs (25). We performed heat map visuals of putative TFs in spermatogonia using the ComplexHeatmap R package.

### 2.10 Peak calling and motif analysis

Peak calling was performed using the CallPeaks function of MACS2 software (https://github.com/taoliu/MACS, v2.1.2.20160309). In detail, the number of translocation events per base pair in the genome was first counted. Next, a smooth profile of these events was produced with a 401-bp moving window around each base pair. Finally, the signal threshold was set at the ratio of 1/5 to determine whether the region is a peak signal or a noise. The default threshold of the q value used by MACS2 was 0.05. Differentially accessible peaks among different cell types were identified using the FindMarkers function of the Signac package. CoveragePlot function was used to visualize genome browser views of Wnt pathway genes. JASPAR2020 (https://jaspar.genereg.net/) database was used to index motif information of TFs. TFs motif activities were computed using the chromVar package. Motif analysis was carried out with the FindMotifs function of the Signac package. Motifs with p-values <0.05 were visualized using the MotifPlot function.

### 2.11 Spatial transcriptomic analysis

We collected marker genes of each cell type from previous research (15). Then, we calculated the cell type scores of each voxel with the AUCell method according to these marker genes. By applying the SpatialFeaturePlot function of Seurat, we visualized the cell type score of each voxel. Similarly, we collected a gene set of hallmark Wnt signaling from MsigDB. We visualized the Wnt signaling using the SpatialFeaturePlot function.

### 2.12 Proteomics data analysis

Proteomics data of NOA (n = 9) and normal (n = 9) samples were downloaded as raw profile data (.raw) from a previous study **(** **Supplementary Table S5**) (26). Proteomics data analysis was performed using Progenesis QI for Proteomics version 4.1 (Nonlinear dynamics, Waters Corporation, Milford, MA, USA). Raw profile data (.raw) of the 18 samples were imported into Progenesis QI for Proteomics with low energy and high energy threshold set to auto and data lock mass-corrected. Next, normalization of the protein abundances was performed utilizing the default method “Normalize to all proteins” by which protein amounts in individual runs are normalized to one run automatically selected as the normalization reference.

### 2.13 Statistical analysis

All statistical analyses were compiled in R (v4.1.0). Student’s *t*-test was conducted for comparison between the two groups. ANOVA was used to determine the significance among three or more than three groups. Correlation coefficients were computed using Pearson’s correlation. Parameters were defaulted if there was no indication. p < 0.05 was considered significant. *p < 0.05, **p < 0.01, ***p < 0.001, and ****p < 0.0001.

## 3 Results

### 3.1 Identification of a hub gene module expressing Wnt signaling in NOA

**Figure 1:**
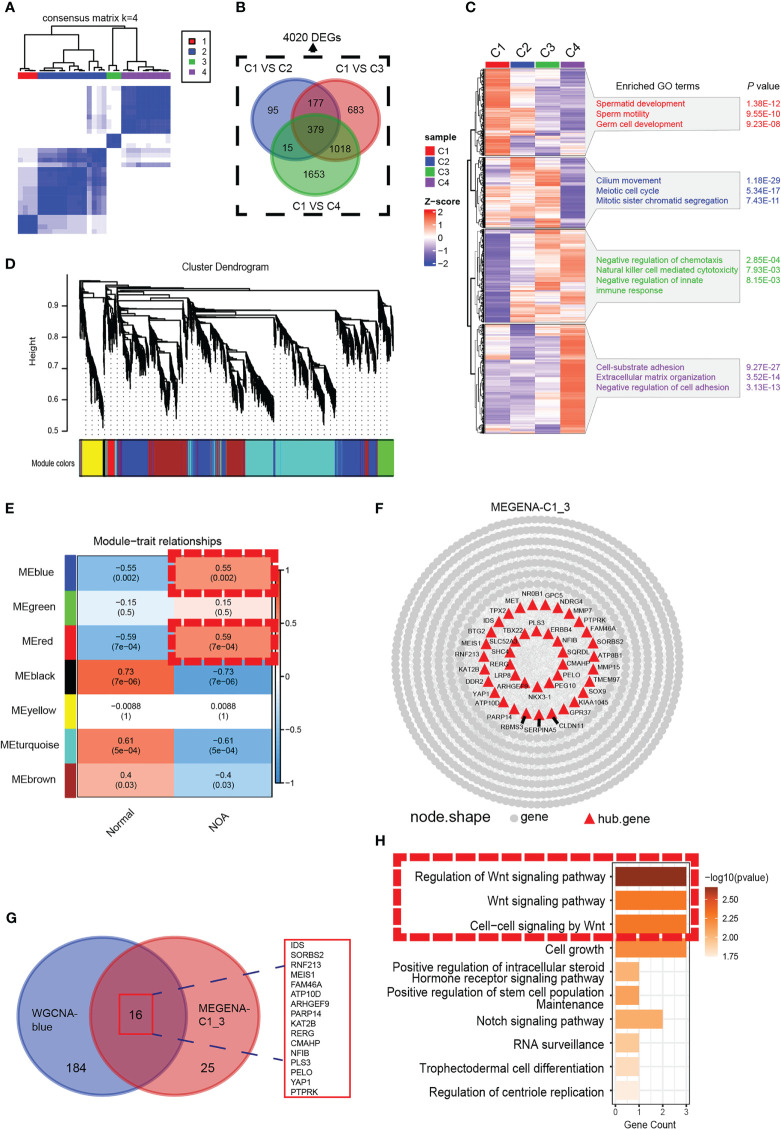
Identification of a NOA-specific hub gene module expressing Wnt signaling. **(A)** The 29 samples were split into four clusters by the consensus clustering matrix (k = 4). All of the normal samples were just categorized into cluster 1. **(B)** Venn diagram was used to preserve 4,020 genes specific to NOA. **(C)** Enriched GO terms and p-value of four clusters. **(D)** The cluster dendrogram of 4,020 differentially expressed genes. **(E)** The heat map shows the relationship between gene modules and clinical characteristics. Each cell contains the corresponding correlation coefficient and p-value. Dashed red squares indicate gene modules highly correlated with NOA. **(F)** Intergenic connectivity of genes in MEGENA_C1_3 and identification of hub genes. **(G)** Venn diagram showing all identified hub genes and their overlap in WGCNA-blue and MEGENA-C1_3. **(H)** GO enrichment analysis was performed on the overlapping genes. Dashed red squares indicate Wnt signaling. NOA, non-obstructive azoospermia; GO, Gene Ontology.

We analyzed the gene expression data of 29 samples (3 normal and 26 NOA) in GSE45885. For further analysis of the heterogeneity of NOA, we conducted unsupervised clustering with the 29 samples (**Figure 1A**). Consequently, we obtained four clusters, with C1 as the normal group and other groups as the NOA group, which not only suggested the transcriptomic difference between normal and NOA samples but also revealed the heterogeneity within different NOA samples, which were further substantiated by principal component analysis (PCA) (**Figure S1A**). GO enrichment analysis showed unique enriched GO terms of these four clusters, including spermatid development for the C1 group, meiotic cell cycle for the C2 group, immune response for the C3 group, and negative regulation of cell adhesion for the C4 group, which indicated substantial heterogeneity within NOA (**Figure 1C**). In order to precisely identify the difference in molecular pathways among these clusters that we accessed, setting the cutoff criterion as |log2(FC)| > 1 and p < 0.01, we identified 4,020 differentially expressed genes (DEGs) (**Figure 1B**). We intersected gene modules based on DEGs calculated by WGCNA and MEGENA algorithms to determine hub modules highly expressed in NOA. In WGCNA, we divided 4,020 DEGs into seven gene modules (**Figure 1D**; **Figure S1B**). The heat map shows that the blue and red gene modules are highly correlated with NOA (**Figure 1E**). In the MEGENA, we identified 242 modules in 4,020 DEGs, and we found a large degree of overlap between the blue module of WGCNA and the MEGENA-C1_3 module (**Figures 1F, G**). Further, we demonstrated the expression levels of these 16 overlapping genes to be upregulated in NOA samples (**Figure S1C**). Subsequently, we found that the Wnt pathway was enriched in the hub gene module, which suggested potential biological functions to induce the occurrence and promotion of NOA (**Figure 1H**).

### 3.2 Landscape of scRNA-seq of NOA

**Figure 2:**
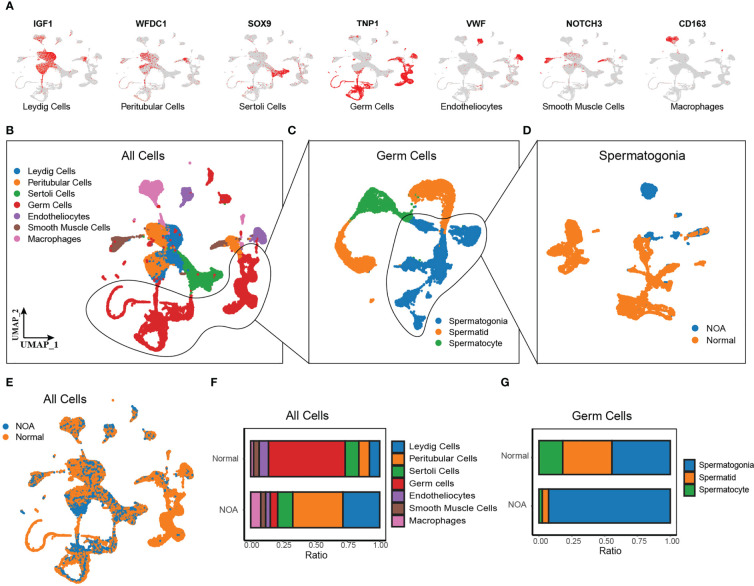
Single-cell RNA-seq of NOA and normal samples. **(A)** UMAP plots of known marker genes of each major cell type. **(B–D)** UMAP plots of all cells from 29 NOA and normal samples (left panel), germ cells (middle panel), and spermatogonia (right panel). Colored by cell types. **(E)** UMAP plots of all cells, colored by tissue origin. **(F)** The proportions of each cell cluster in NOA and normal samples. **(G)** The proportions of each germ cell cluster in NOA and normal samples. NOA, non-obstructive azoospermia; UMAP, uniform manifold approximation and projection.

Additionally, we comprehensively investigated the biological significance of Wnt signaling in NOA at single-cell resolution. We obtained the scRNA-seq data of 29 samples (7 NOA and 22 normal samples) from Nie et al. (13). and Zhao et al. (14). After quality control, normalization, and removal of the batch effect, we detected a total of 61,622 cells colored by sample origins (**Figure S2A**). According to markers from previous research (15), we identified seven cell types using the UMAP method (**Figures 2A, B**), including Leydig cells (expressing IGF1), peritubular cells (expressing WFDC1), Sertoli cells (expressing SOX9), germ cells (expressing TNP1), endotheliocytes (expressing VWF), smooth muscle cells (expressing NOTCH3), and macrophages (expressing CD163). Cells from NOA and normal samples were well-distributed, indicating the removal of the batch effect (**Figure 2E**). The proportion of each cell type shows a large variation in the proportion of Leydig cells, peritubular cells, and germ cells between NOA and normal samples (**Figure 2F**). As germ cells have dramatic effects on the etiology of NOA, we further identified the subpopulations of germ cells (**Figure 2C**), including spermatogonia (expressing SMS, ALDH1A1, KIT, and TKTL1), spermatocyte (expressing PIWIL1 and OVOL2), and spermatid (expressing PRM3, ACR, TEX29, and ACRV1) (**Figures S2B–D**). Cell type proportion plot illustrates high proportion of spermatogonia whereas low proportion of spermatocyte and spermatid in NOA (**Figure 2G**). To determine whether this was due to failure of spermatogonia differentiation, we extracted spermatogonia for further downstream analysis (**Figure 2D**).

### 3.3 Dysfunctional Wnt signaling pathway of spermatogonia in NOA

**Figure 3:**
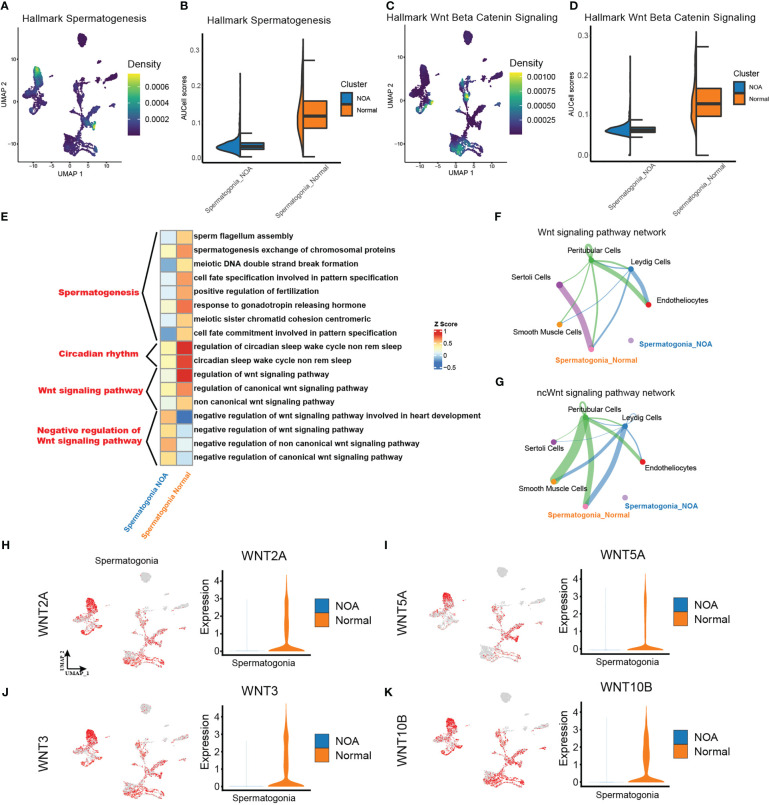
Different strengths of Wnt signaling pathway exist within spermatogonia. **(A, C)** Density scatterplots of hallmark spermatogenesis gene set (left panel) and of hallmark Wnt beta-catenin signaling gene set (right panel) in spermatogonia. **(B, D)** Half-violin plots showing the AUCell scores of spermatogenesis gene set (left panel) and of Wnt signaling gene set (right panel) in spermatogonia between NOA and normal samples. **(E)** Enriched GO terms of spermatogonia. **(F, G)** The inferred signaling pathway between the spermatogonia and other cell types. WNT signaling pathway (upper panel). Non-canonical Wnt (ncWnt) signaling pathway (lower panel). The line thickness is proportional to the number of ligand–receptor pairs. **(H–K)** UMAP plots show activities of the Wnt pathway genes in spermatogonia (left panel). Violin plots show the expression of corresponding genes in spermatogonia across NOA and normal samples. NOA, non-obstructive azoospermia; GO, Gene Ontology; UMAP, uniform manifold approximation and projection.

After extracting spermatogonia, we scored the activities of spermatogenesis and Wnt signaling from MsigDB hallmark gene sets (http://www.gsea-msigdb.org/gsea/msigdb) using AUCell. In line with the theoretical expectation, spermatogonia in NOA had a lower spermatogenesis score (**Figures 3A, B**). Separately, we found a lower Wnt signaling score in the spermatogonia of NOA (**Figures 3C, D**). Then, to identify the potential role of downregulated Wnt signaling in NOA, we performed GO analysis, and the results suggested that the negative regulation of the Wnt signaling pathway was more intense in NOA than in normal (**Figure 3E**). Surprisingly, we also found that circadian rhythms were different in NOA and normal. Next, we explored cell-type interactions through the Wnt signaling pathway using CellChat. CellChat predicted that there were more Wnt signaling interactions in normal spermatogonia (**Figures 3F, G**). Finally, we tested the expression of Wnt pathway genes. The results display decreasing expression of Wnt pathway genes in spermatogonia of NOA (**Figures 3H–K**). Taking these results together, we suggest that there is a dysfunctional Wnt signaling pathway in the spermatogonia of NOA. To assess the Wnt signaling pathway at the protein level, we download proteomics data of NOA (n = 9) and normal (n = 9) samples from a previous study (26). We tested the expression of two proteins related to Wnt signaling (WNT3 and WNT5A). The results displayed decreasing expression of WNT3 and WNT5A proteins in NOA samples (**Figure S4A**).

### 3.4 Identification of key genes correlated with dysfunctional Wnt signaling in spermatogonia of NOA

**Figure 4:**
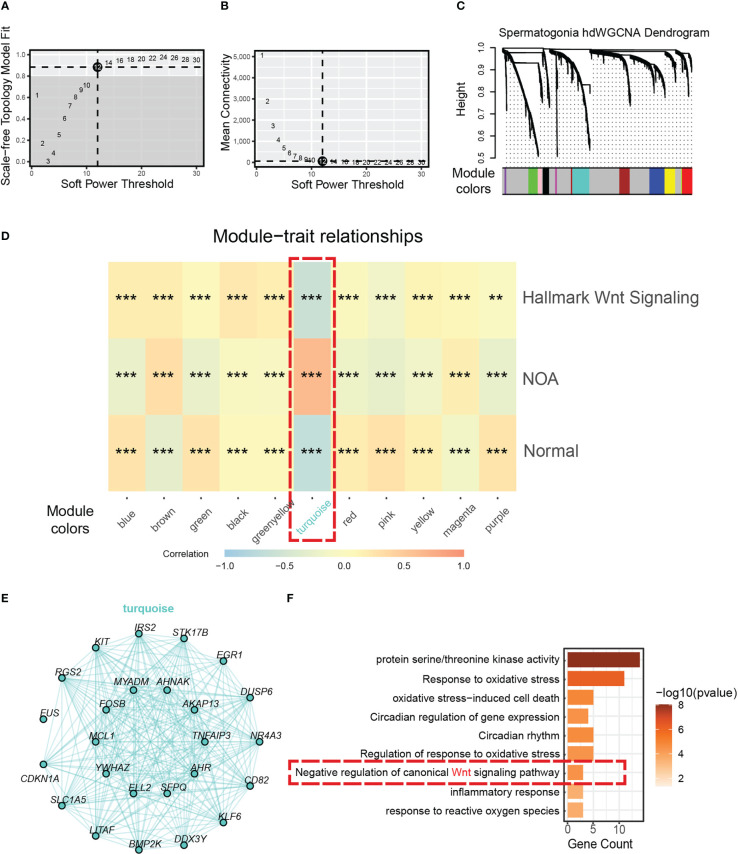
Identification of a hub gene module correlated with NOA in spermatogonia. **(A)** The relationship between the scale-free fit index and various soft-thresholding powers. **(B)** The relationship between the mean connectivity and various soft-thresholding powers. **(C)** The cluster dendrogram of all genes in spermatogonia. Branches of the cluster dendrogram of the most connected genes gave rise to 11 gene co-expression modules. **(D)** Heat map of the correlation between module eigengenes and phenotype (normal, NOA, and hallmark Wnt signaling score). Dashed red square indicates gene module correlated with NOA (*<0.05, **<0.01, ***<0.001, and ****<0.0001). **(E)** Intergenic connectivity of genes in turquoise module. **(F)** GO enrichment analysis was performed on the genes of turquoise module. Dashed red square indicates negative regulation of Wnt signaling. NOA, non-obstructive azoospermia; GO, Gene Ontology.

To discover the key genes driving dysfunctional Wnt signaling in NOA, we used hdWGCNA, which was designed specifically for scRNA-seq to find core genes. By using the soft threshold power of 12 (scale-free R^2 = ^0.85) (**Figures 4A, B**), we divided genes in spermatogonia into 11 gene modules (**Figure 4C**). Then, we applied correlation analysis to find the biological correlation between gene modules and important traits. Fortunately, we used a gene module for our correlation analysis—the turquoise module. The turquoise module had a significantly high positive relationship with NOA and the hallmark of Wnt signaling (**Figure 4D**). The top 25 genes with the highest correlation degree in the turquoise module are exhibited in **Figure 4E**. To determine the function that this gene module may be involved in, we carried out GO annotation. The GO annotation identified functions, which include response to oxidative stress, circadian rhythm, and inflammatory response (**Figure 4F**).

### 3.5 Landscape of scATAC-seq of normal testis

**Figure 5:**
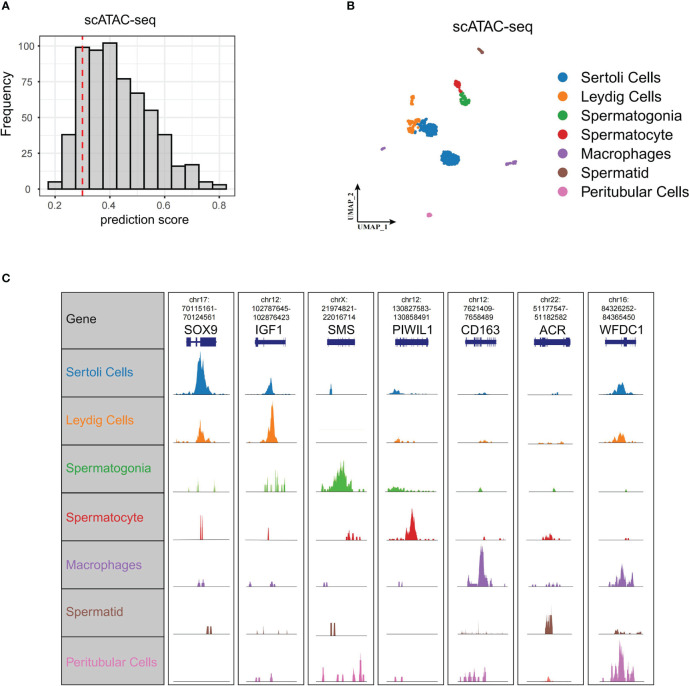
ScATAC-seq of normal testis. **(A)** Cell number of different scATAC-seq cell prediction scores displayed by bar plot. We set 0.3 as threshold (red dashed line) to select cells for the following analysis. **(B)** UMAP of scATAC-seq data showing all the high-quality filtered single cells. **(C)** Genome browser views of scATAC-seq for the marker genes in scRNA-seq. scATAC-seq, single-cell assay for transposase-accessible chromatin sequencing; UMAP, uniform manifold approximation and projection.

To further delineate the regulatory network controlling Wnt signaling in spermatogenesis, we profiled scATAC-seq of normal testis from Garcia-Alonso et al. (15). We predicted cell types of scATAC-seq based on anchor points of our former scRNA-seq. We isolated 619 cells from 750 cells with prediction scores greater than 0.3 (**Figure 5A**). These 619 cells and their corresponding cell types, including Leydig cells, peritubular cells, Sertoli cells, spermatogonia, spermatocyte, spermatid, and macrophages, are shown in **Figure 5B**. We did not identify endotheliocytes and smooth muscle cells. The genome browser views of known marker genes (SOX9 for Sertoli cells, IGF1 for Leydig cells, SMS for spermatogonia, PIWIL1 for spermatocyte, ACR for spermatid, CD163 for macrophages, and WFDC1 for peritubular cells) demonstrated the accuracy of cell type annotation (**Figure 5C**).

### 3.6 Transcription factors that regulate Wnt signaling pathway

**Figure 6:**
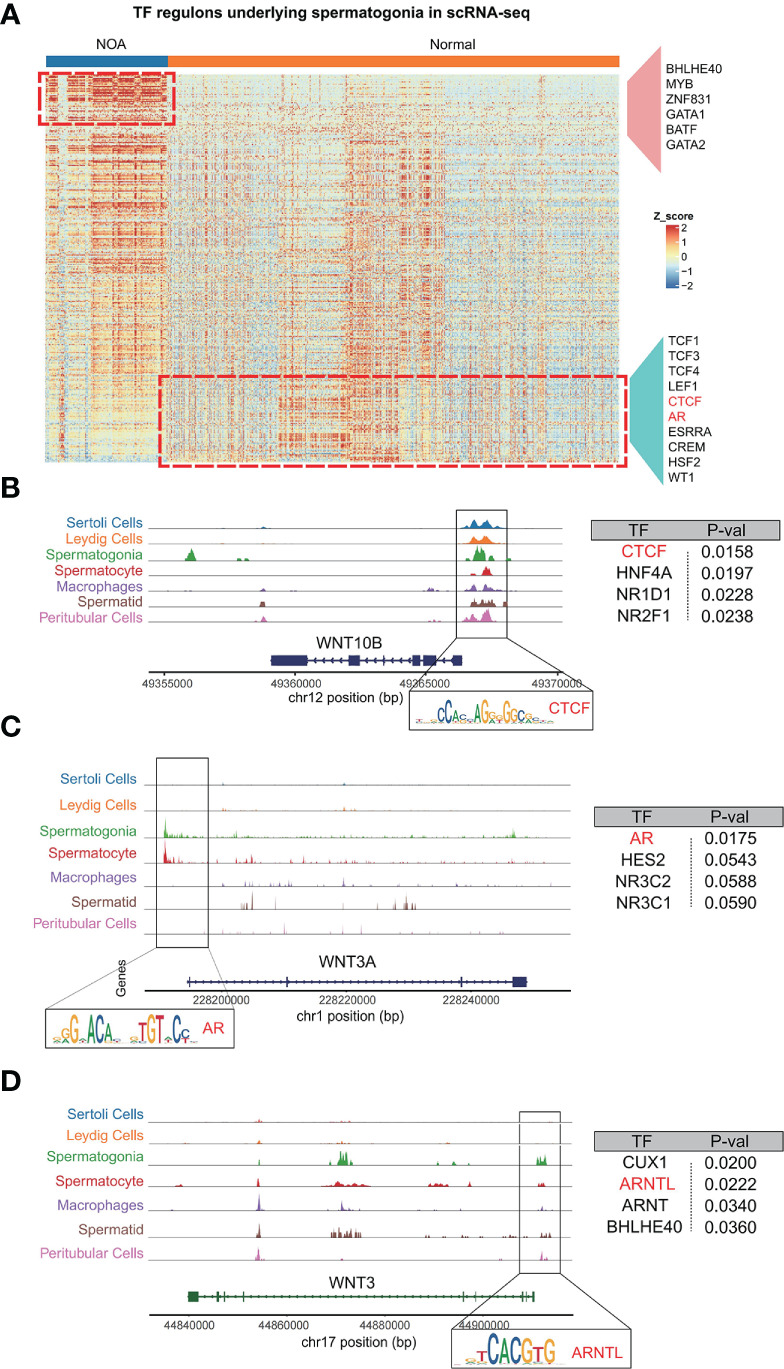
Analysis of relationships among spermatogonia, Wnt pathway genes, and TFs (CTCF, AR, and ARNTL). **(A)** The heat map shows the inferred activities of TFs in spermatogonia underlying NOA and normal samples. **(B–D)** Regions of enriching peaks upstream of WNT10B (Chr12) **(B)**, WNT3A (Chr1) **(C)**, and WNT3 (Chr17) **(D)**. Motifs located in this region are annotated. Summary table for the annotated motifs is shown on the right. TFs, transcription factors; NOA, non-obstructive azoospermia.

Having constructed scATAC-seq of the normal testis, we set out to find transcription factors that regulate Wnt signaling. We first speculated putative transcription factors in spermatogonia with pySCENIC. As expected, spermatogonia in NOA and normal samples were driven by respective TFs, including BHLHE40, MYB, ZNF831, GATA1, and BATF for NOA spermatogonia, and TCF1, LEF1, CTCF, AR, and ESRRA for normal spermatogonia (**Figure 6A**). Consistently, a previous study has shown that TCF1 and LEF1 function to maintain the activation of Wnt signaling (27). We then focus on the relationships between these inferred transcription factors and Wnt signaling. Based on scATAC-seq of the normal testis, we found Wnt pathway genes, like WNT10B, WNT3A, and WNT3, to be specifically and prominently expressed in spermatogonia (**Figures 6B–D**). Further analysis revealed that motifs were found with high accessibility enriched in the upstream region close to Wnt pathway genes. We uncovered TFs (like CTCF, AR, and ARNTL) as the enriched motifs (**Figures 6B–D**). Coincidentally, we also found the same TFs (CTCF and AR) using pySCENIC in scRNA-seq (**Figure 6A**). Thus, our scATAC-seq confirmed the reliability of these TFs generated from pySCENIC. In humans, CTCF, as a zinc-finger DNA-binding protein, is required to activate meiotic genes for a complete spermatogenesis program (28). AR (known as androgen receptor) plays an unmistakable role in the development of male reproductive functions (29). ARNTL is a critical transcription factor controlling circadian rhythm (30). Our study is the first to use single-cell multi-omics analysis to find if these TFs (CTCF, AR, and ARNTL) exert functions in the normal spermatogonia, probably by maintaining the Wnt pathway. The single-cell multi-omics analysis is advantageous for the comprehensive understanding of the role of these TFs in normal spermatogonia. Here, for the first time, we found that these TFs (CTCF, AR, and ARNTL) exert functions in the normal spermatogonia, probably by maintaining the Wnt pathway.

### 3.7 Expression of CTCF, AR, and ARNTL in NOA samples

**Figure 7:**
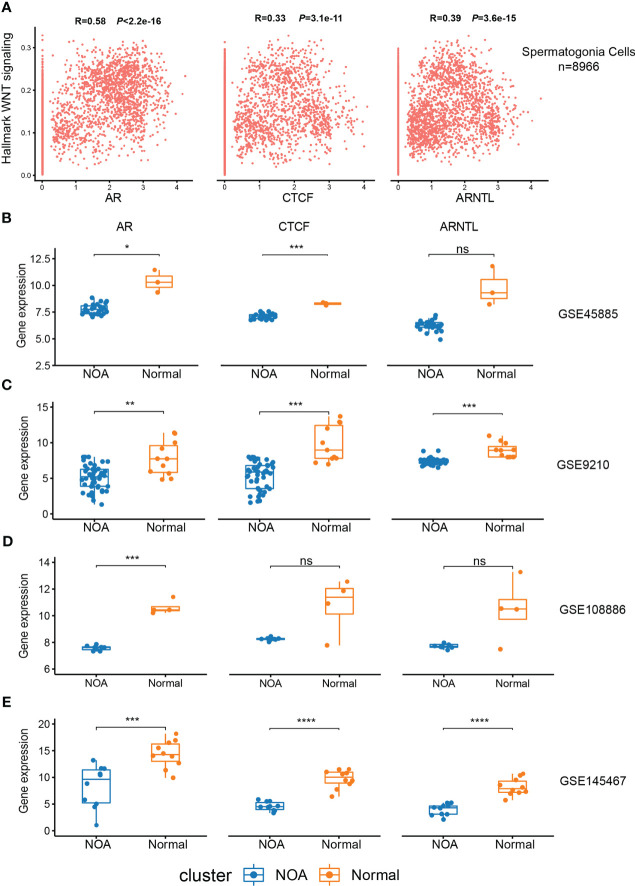
Expression of CTCF, AR, and ARNTL in scRNA-seq and RNA-seq. **(A)** The relationship between the expression of three TFs and hallmark Wnt signaling in spermatogonia of scRNA-seq. **(B–E)** Expression of three TFs between NOA and normal samples in GSE45885 **(B)**, GSE9210 **(C)**, GSE108886 **(D)**, and GSE145467 **(E)**. (*<0.05, **<0.01, ***<0.001, and ****<0.0001) and those with p-values >0.05 as not significant “ns”. ScRNA-seq, single-cell RNA sequencing; RNA-seq, RNA sequencing; TFs, transcription factors; NOA, non-obstructive azoospermia.

In order to validate the association between the expression of CTCF, AR, and ARNTL and Wnt signaling in spermatogonia, we performed a correlation analysis between the activity of Wnt signaling and the expression of these three TFs using the FeatureScatter function in Seurat. The results showed that the levels of CTCF, AR, and ARNTL were significantly positively correlated with hallmark WNT signaling (p < 2.2e−16, p < 3.1e−11, and p < 3.6e−15, respectively) (**Figure 7A**). Next, we collected five RNA-seq datasets to compare the expression of three TFs in NOA and normal samples. The downward trend was pronounced in NOA samples (**Figures 7B–E**; **S3A**). As previously mentioned, we found that these three TFs exert functions in the normal spermatogonia (**Figures 6A–D**). Consistent with this finding, here, we concluded that low expression of CTCF, AR, and ARNTL correlates with NOA.

### 3.8 The cell type spatial distribution of Wnt signaling

**Figure 8:**
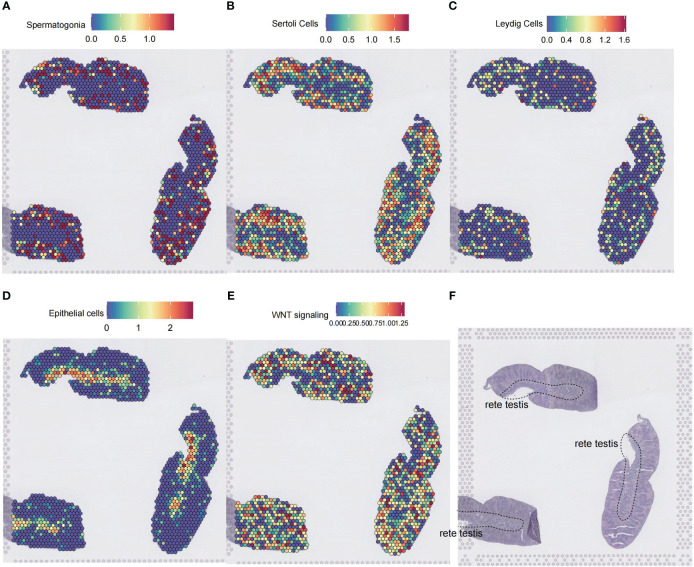
Spatial transcriptomic analysis of Wnt signaling in the testis. **(A–D)** Cell type inference of spatial voxels from three consecutive spatial transcriptomics slides of a 14 postconceptional week testis. Gene expressions in the dorsolateral prefrontal cortex were profiled from 10-μm serial tissue sections by the 10X Genomics Visium platform. Color intensity is proportional to cell type-specific gene expression in the scRNA-seq dataset. **(E)** The spatial voxels enriched for the Wnt signaling. For each spatial voxel, the enrichment score was calculated by the GSEA method, which tested whether the Wnt signaling was enriched in the highly expressed genes. **(F)** The corresponding histology testis slide. ScRNA-seq, single-cell RNA sequencing; GSEA, gene set enrichment analysis.

To further illustrate the spatial localization of Wnt signaling, we used a spatial transcriptomics dataset of testis from Garcia-Alonso et al. (15). The dataset contains three consecutive spatial transcriptomics slides of the normal testis. By applying the SpatialFeaturePlot function of Seurat, we annotated cell types by calculating marker gene scores for cell types of interest with the AUCell method. Rete testis is comprised mainly of epithelial cells (**Figures 8D, F**). Spermatogonia, Sertoli cells, and Leydig cells were distributed at the periphery of the rete testis (**Figures 8A–C**). Spatial localization of the Wnt signaling-enriched voxels resembled the distribution patterns of spermatogonia, Sertoli cells, and Leydig cells on the slide (**Figure 8E**).

## 4 Discussion

The role of spermatogonia for reproductive function is like the role of fuel for rockets, providing a constant stream of spermatocytes to differentiate into spermatozoa (31). Although an increasing body of evidence suggested the essential role of spermatogonia in the complete spermatogenic process, the alteration in cellular signaling pathways of spermatogonia in the context of NOA remains unknown. Wnt signaling is an evolutionally conserved signaling pathway playing a critical role in development and disease. A previous study has implicated the significant role of the Wnt signaling pathway in multiple stages of spermatogenesis (32). However, Wnt signaling activity in NOA patients has not been described previously. In this study, we explored the Wnt signaling activity of normal and NOA spermatogonia in a multi-omics approach.

Our research suggested that the Wnt signaling pathway is significantly enriched in the hub gene module in the occurrence of NOA by analyzing bulk RNA-seq. By performing WGCNA and MEGENA to the DEGs between normal and NOA samples, we obtained the hub gene module related to NOA. GO analysis of the hub gene module showed enrichment of the Wnt signaling pathway. Moreover, we found that the Wnt signaling pathway is dysregulated in spermatogonia of NOA by analyzing scRNA-seq. ScRNA-seq allowed us to precisely distinguish cell types at an unparalleled resolution. We collected gene sets of spermatogenesis, the Wnt signaling pathway, and other GO biological processes, and we employed AUCell and GSVA to calculate the enrichment of these gene sets for single cells according to the gene expression on single cells. The dysregulated pathways in spermatogonia of NOA include spermatogenesis, circadian rhythm, and Wnt signaling pathway. This is consistent with the findings of bulk RNA-seq. Other important findings include key TFs of spermatogonia in patients with NOA. Although the possible causes of dysregulated pathways in spermatogonia of NOA patients are unknown, finding key TFs to improve normal spermatogonia might still be useful for treating the disease. By analyzing scATAC-seq, we identified TFs (CTCF, AR, and ARNTL), which were enriched in spermatogonia. Surprisingly, the CoveragePlot function of scATAC-seq indicated that these TFs can be involved in the Wnt signaling pathway. While we had no scATAC-seq data regarding NOA patients, we also found significantly different expressions of these three TFs in NOA by RNA-seq.

Previous studies have demonstrated that these three TFs played a pivotal role in Wnt signaling and spermatogenesis. CTCF, as one of these three TFs, is an upstream regulator implicated in lineage-specific gene regulation (33). Moreover, Wnt signaling in colon cancer requires CTCF binding to oncogenic super-enhancers (34). AR is a transcription factor closely related to spermatogenesis. There are many pieces of evidence suggesting that AR can invoke dynamic changes in the Wnt signaling function including nuclear cotrafficking and transrepression (35). ARNTL, also referred to as BMAL1, is a key regulator of the circadian clock network that imparts temporal regulation to diverse biological processes including spermatogenesis (36). Guo et al. demonstrated that attenuation of ARNTL function resulted in the downregulation of genes in the Wnt pathway (37). However, single-cell multi-omics analysis has never been used in these previous studies. The single-cell multi-omics analysis is beneficial for enhancing a comprehensive understanding of cellular events. Our study is the first to use single-cell multi-omics analysis to find if these TFs (CTCF, AR, and ARNTL) were involved in the regulation of spermatogonia in NOA patients, possibly by regulating Wnt signaling.

Overall, in our study, we found dysfunctional Wnt signaling of spermatogonia in NOA, and three TFs may be involved in this dysfunctional Wnt signaling using multi-omics analysis. These findings provide a new mechanism for NOA and new therapeutic targets for NOA patients. However, there are limitations to our study. Sequencing data cannot reveal all the details of dysfunctional Wnt signaling in spermatogonia of NOA. Molecular experiments, such as immunohistochemistry experiments or gene knockdown experiments, are required. In addition, further studies to prove that these TFs can regulate Wnt signaling to effectively treat NOA are needed.

## 5 Conclusion

NOA is a desperate and devastating disease for men who have a desire for fertility. We obtained RNA-seq, scRNA-seq, scATAC-seq, and spatial transcriptomic data of normal and NOA samples. We found dysfunctional Wnt signaling of spermatogonia in NOA, and three TFs may be involved in this dysfunctional Wnt signaling. Collectively, these findings provide a new mechanism for NOA and new therapeutic targets for NOA patients.

## Data availability statement

The datasets presented in this study can be found in online repositories. The names of the repository/repositories and accession number(s) can be found in the article/**Supplementary Material**.

## Ethics statement

All the gene expression data and clinical information used in this study have been approved by the Ethics Committees at the corresponding public institutions.

## Author contributions

SZ and CL conceptualized and designed the study. SZ and LC collected the data. SZ and XL analyzed data. SZ and HT wrote and contributed to the manuscript. LC and XL revised the manuscript. All authors contributed to the article and approved the submitted version.
