# Supplementary figures and images for: Single-cell multi-omics analysis reveals dysfunctional Wnt signaling of spermatogonia in non-obstructive azoospermia
Source: Front Endocrinol (Lausanne). 2023 Jun 6;14:1138386. doi: 10.3389/fendo.2023.1138386 (PMC10273265; doi:10.3389/fendo.2023.1138386)

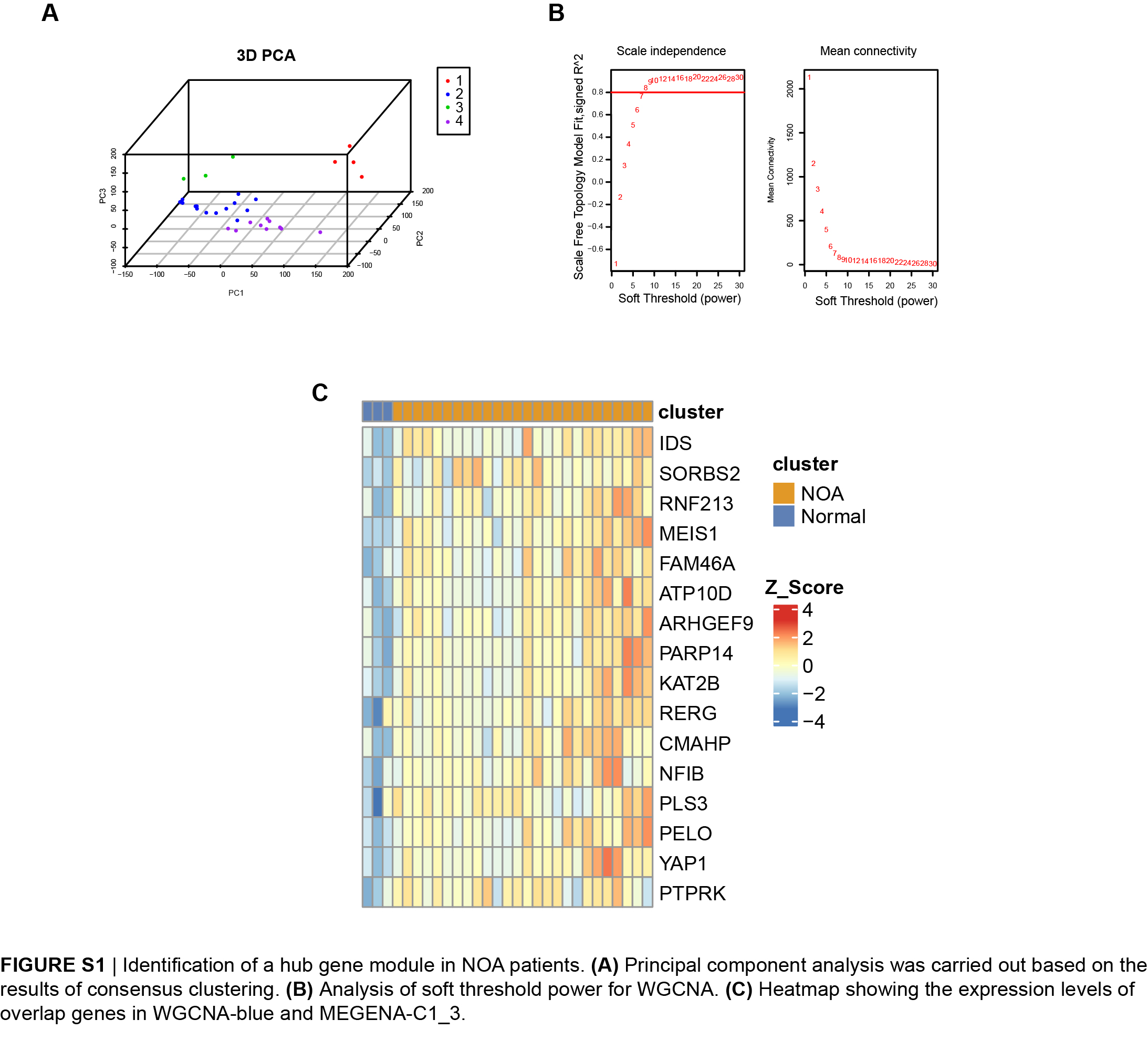

Supplement: Supplementary file 1 [file Image_1.jpeg]

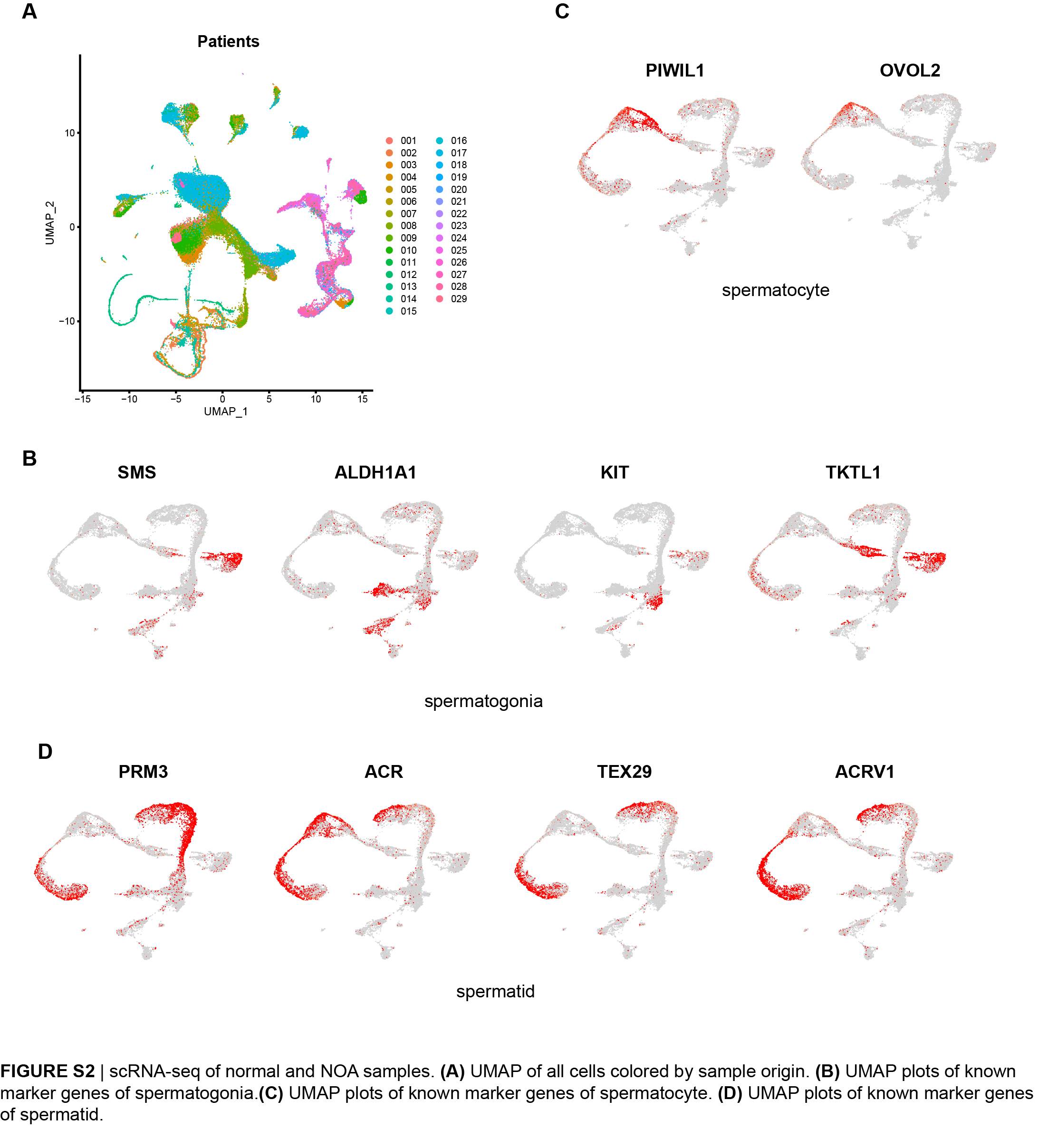

Supplement: Supplementary file 2 [file Image_2.jpeg]

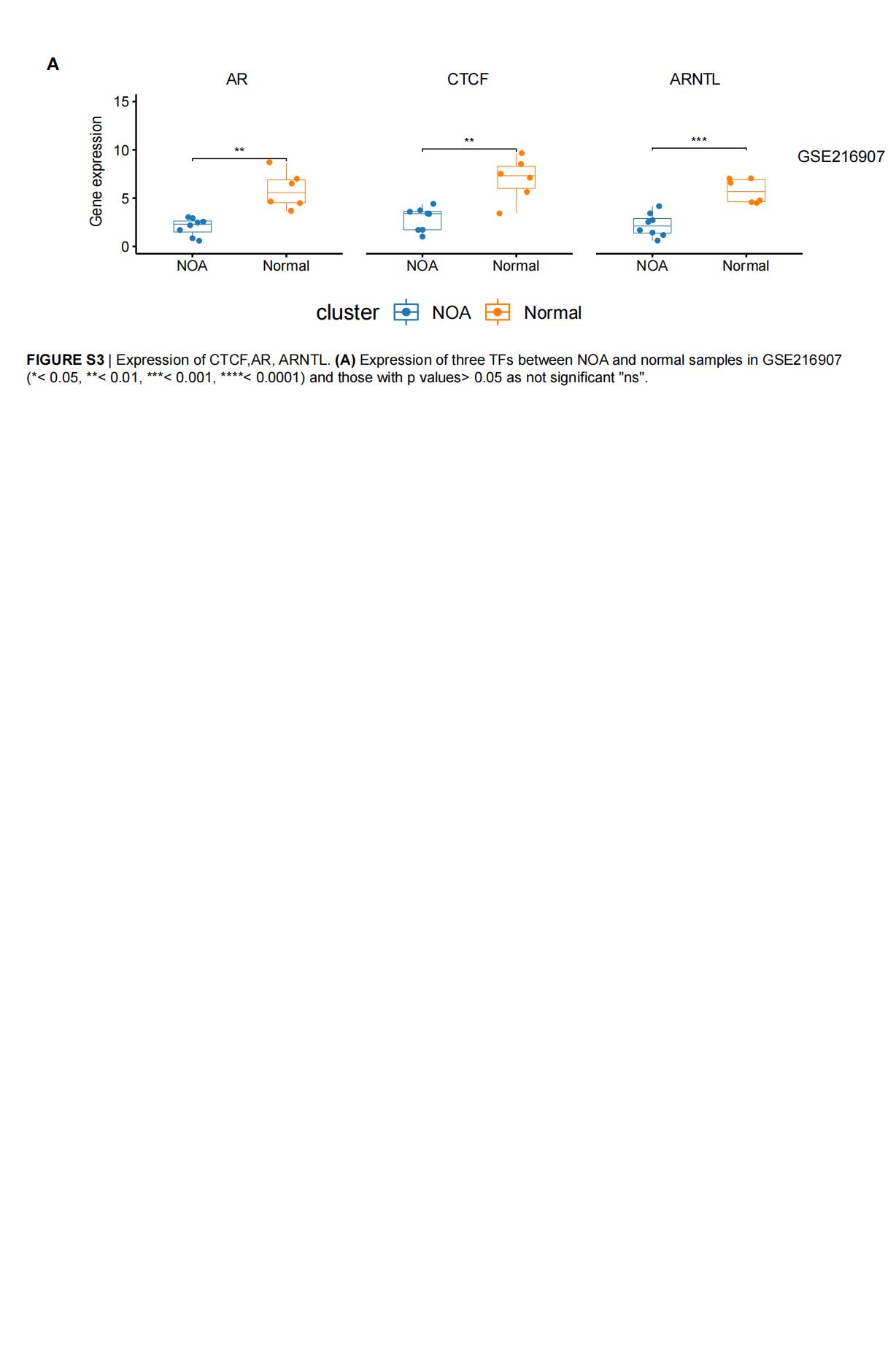

Supplement: Supplementary file 3 [file Image_3.jpeg]

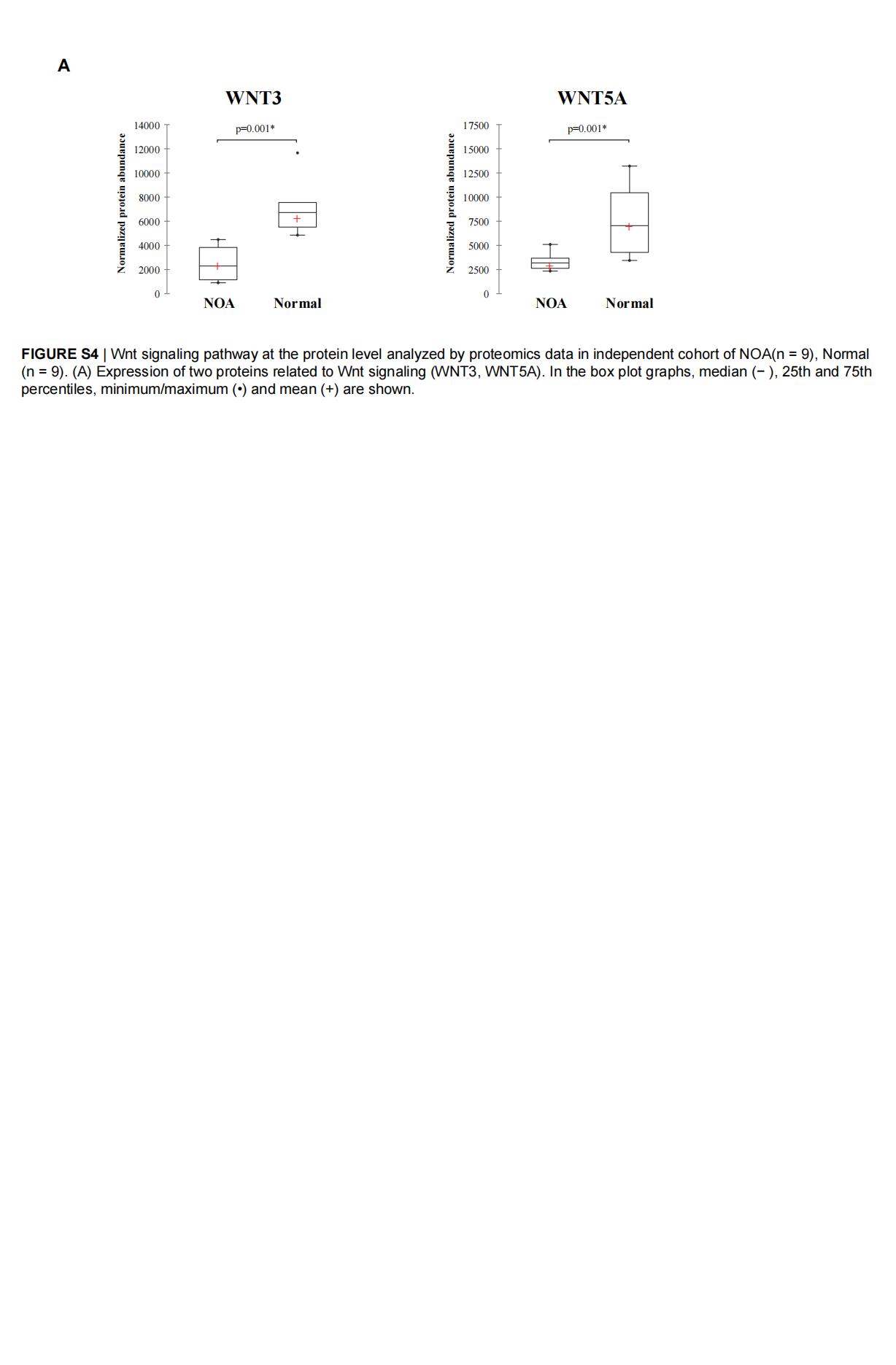

Supplement: Supplementary file 4 [file Image_4.jpeg]
